# An Integrated District Mapping Strategy for Loiasis to Enable Safe Mass Treatment for Onchocerciasis in Gabon

**DOI:** 10.4269/ajtmh.21-0799

**Published:** 2021-11-15

**Authors:** Sylvie Ntsame Ella, Kisito Ogoussan, Katherine Gass, Lee Hundley, Peter J. Diggle, Olatunji Johnson, Marco Biamonte, Julienne Atsame

**Affiliations:** ^1^ Programme de Lutte Contre les Maladies Parasitaires, Ministère de la Santé du Gabon, Libreville, Gabon;; ^2^ FHI 360, Washington, District of Columbia;; ^3^ Neglected Tropical Diseases Support Center, Task Force for Global Health, Decatur, Georgia;; ^4^ CHICAS Research Group, Lancaster Medical School, Lancaster University, Bailrigg, Lancaster, United Kingdom;; ^5^ Drugs and Diagnostics for Tropical Diseases, San Diego, California

## Abstract

The lack of a WHO-recommended strategy for onchocerciasis treatment with ivermectin in hypo-endemic areas co-endemic with loiasis is an impediment to global onchocerciasis elimination. New loiasis diagnostics (LoaScope; Loa antibody rapid test) and risk prediction tools may enable safe mass treatment decisions in co-endemic areas. In 2017–2018, an integrated mapping strategy for onchocerciasis, lymphatic filariasis (LF), and loiasis, aimed at enabling safe ivermectin treatment decisions, was piloted in Gabon. Three ivermectin-naïve departments suspected to be hypo-endemic were selected and up to 100 adults per village across 30 villages in each of the three departments underwent testing for indicators of onchocerciasis, LF, and loiasis. An additional 67 communities in five adjoining departments were tested for loiasis to extend the prevalence and intensity predictions and possibly expand the boundaries of areas deemed safe for ivermectin treatment. Integrated testing in the three departments revealed within-department heterogeneity for all the three diseases, highlighting the value of a mapping approach that relies on cluster-based sampling rather than sentinel sites. These results suggest that safe mass treatment of onchocerciasis may be possible at the subdepartment level, even in departments where loiasis is present. Beyond valuable epidemiologic data, the study generated insight into the performance of various diagnostics and the feasibility of an integrated mapping approach utilizing new diagnostic and modeling tools. Further research should explore how programs can combine these diagnostic and risk prediction tools into a feasible programmatic strategy to enable safe treatment decisions where loiasis and onchocerciasis are co-endemic.

## INTRODUCTION

Currently, there is no WHO-recommended strategy to eliminate onchocerciasis in countries endemic for *Loa loa* because of the concern about serious adverse events (SAEs) that may occur when ivermectin (IVM) is given as part of mass drug administration (MDA) to individuals with high-intensity (> 20,000 mf/mL) *L. loa* infections.^1^^,^^2^ This represents a substantial obstacle in the global effort to eliminate onchocerciasis.^3^ In *L. loa* co-endemic areas where onchocerciasis is classified as “meso- “or “hyper-endemic” (nodule rate ≥ 20% and/or microfilaria prevalence ≥ 40%), IVM treatment was recommended despite the heightened risk of SAEs, due to the benefit of treatment in preventing blindness.^4^ Strategies for areas where onchocerciasis is “hypo-endemic” (nodule rate < 20% and/or microfilaria prevalence < 40%) and the risk of blindness is much less, however, require more care in making treatment decisions, given that the prevalence of loiasis (and high-intensity infections, in particular) may vary widely within countries or even within health districts. There is therefore a need for a comprehensive, safe, and cost-effective strategy to eliminate onchocerciasis in IVM-naive areas where *L. loa* is co-endemic that takes into account variation in risk of SAEs across geographic areas.

In recent years, a variety of diagnostic and mathematical tools have been developed that can be incorporated into a strategy aimed at making treatment decisions for onchocerciasis in areas co-endemic with loiasis. The LoaScope (UC Berkeley), a mobile diagnostic method first validated in the field in 2015, can be used to effectively diagnose the presence and intensity of *L. loa* infection using magnified imaging of whole blood.^5^ Use of this rapid, low-cost technology at the point of care allows delivery of IVM only to patients with *L. loa* below a well-documented risk threshold through a “Test-and-Not-Treat” (TaNT) strategy.^1^ However, the level of time and human resources required to implement the TaNT strategy may make it costly and impractical across the entire region where *L. loa* is endemic. At the village level, a mathematical model developed by a team at the Center for Health Informatics, Computing, and Statistics at Lancaster University allows neglected tropical disease (NTD) programs to predict whether the level of risk is below a policy-relevant threshold and it is safe to deliver MDA. This model relies on the positive correlation between community-level prevalence and intensity of infection to predict the proportion of individuals in a given community whose parasite count is likely to exceed a designated threshold for SAE risk.^6^ Practically, this community-level risk model relies on sampling a small number of individuals (e.g., 50–100 per community) to determine whether the risk of observing individuals with high-intensity infections is sufficiently low (< 5% probability that the prevalence of high-intensity infections in the community exceeds 1%).^6^ This threshold was set by the Mectizan Expert Committee during an Atlanta-based meeting in April 2016, which recommended that the Mectizan Donation Program authorize the donation of IVM for MDA in districts hypo-endemic for onchocerciasis in Loa-endemic countries that fall below this risk threshold.^2^

Finally, the Loa antibody rapid test can be used to detect antibodies to a protein expressed by *L. loa* and indicates a history of infection (not necessarily active infection).^7^ The test is particularly useful given that it can be performed at any time of day, unlike the restrictions for using the LoaScope during the hours of 10 am –2 pm due to the diurnal periodicity of the parasite.^8^ The test is also easy to use in the field, making it ideal for use in mapping and decision-making at the population level.

Gabon represents an ideal setting to pilot the proposed strategy given the presence of onchocerciasis and *L. loa* throughout much of the country. In addition, the endemicity of lymphatic filariasis (LF) in the country is presently unknown, making it a good setting to pilot an integrated mapping approach.^9^ Based on the most recent available data, all of the departments in Gabon, including those selected for the integrated mapping, as well as the additional five departments selected for extended loiasis mapping, were considered high risk for loiasis (prevalence > 40% by RAPLOA).^9^ None of the departments have ever received treatment with IVM because of the high loiasis prevalence and risk of SAEs, which may reduce the likelihood that community programs are established within the framework of the health system in Gabon. The primary objective of this study was to identify a feasible strategy to jointly map for onchocerciasis, LF, and loiasis to allow programs to make an informed decision as to whether it is safe to administer IVM through MDA, or if a more targeted approach is needed.

## METHODS

### Site selection.

**Figure 1.**
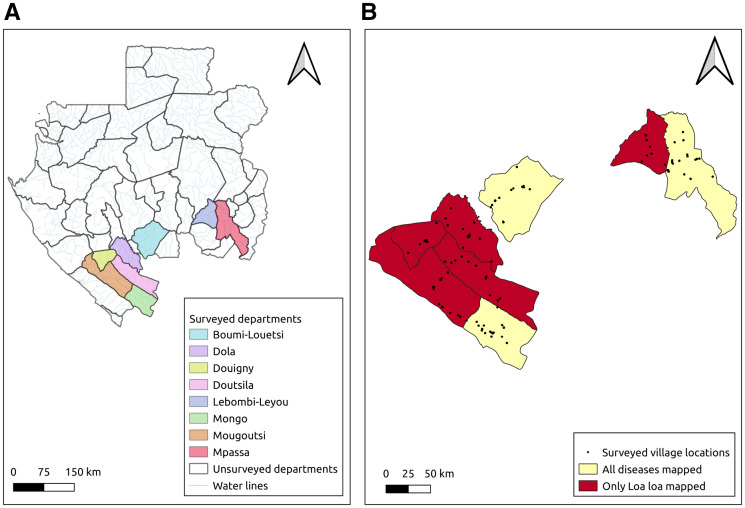
Map of selected departments and villages, southern Gabon.

Three IVM-naive departments (“department” refers to the administrative level 2 boundaries in Gabon, commonly referred to as “districts” in other country contexts) in southern Gabon, Mpassa, Boumi-Louetsi, and Mongo, were selected for the joint mapping of LF, onchocerciasis, and loiasis (Figure 1). All three departments were classified as hypo-endemic for onchocerciasis based on Rapid Epidemiological Mapping of Onchocerciasis (REMO) data.^10^ Within each of the three departments selected for the joint mapping, 30 communities were chosen randomly using probability proportionate to estimated size sampling, based on the most recent census estimates.

Five additional departments (Dola, Douigny, Doutsila, Lebombi-Leyou, and Mougoutsi), surrounding those selected for the joint mapping, were selected to extend only the loiasis mapping. Across the five departments, 67 total communities were strategically selected with input from the Gabonese program. To capture the extent of the spatial correlation in *L. loa* infection and antibody, villages were selected to be 5–10 km apart. By spacing villages in this manner, it enabled validation of the geostatistical modeling predictions regarding the prevalence of high-intensity *L. loa* infections and also created an opportunity to expand the boundary of the areas determined to have a high probability of safe IVM treatment, based on the baseline assessment.

Data collection began December 3, 2017, with the joint mapping work in Boumi-Louetsi, Mpassa, and Mongo, which was completed on December 20, 2017. The additional loiasis-only mapping in Dola, Douigny, Doutsila, Lebombi-Leyou, and Mougoutsi was initiated on November 28, 2018, and completed December 24, 2018.

### Individual selection.

The primary target population for the joint mapping was adults, to align with the current mapping guidance for onchocerciasis.^11^ This target population was extended to include individuals of 10 years old and above to get a better understanding of the age distribution of loiasis. All individuals were required to have been a resident of the community for at least 3 months at the time of selection. The target sample size within each community was 100 individuals; however, because most communities had fewer than 100 individuals ages 10 and above present, sampling ended up being a census of the eligible individuals. In the event that the community had more than 100 individuals present, enrollment stopped once 100 individuals were reached.

To enroll individuals into the study, members of the study team, accompanied by local volunteers, walked through the community, stopping at each house to enroll individuals. Upon arrival at a given household, a member of the study team would describe the purpose of the study, determine the number of eligible members present in the household, and ask each of these members for their consent to participate. Demographic information, including age, sex, and residency, were collected at the time of household enrollment. In addition to capturing basic demographic information, the surveyors asked participants whether they had ever had a worm migrate across their eye and, if so, how many times this had happened in the past year. Although similar to the RAPLOA questionnaire,^12^ the wording and number of questions used in this version of the questionnaire represented a slight departure from the standard questionnaire. Participants were also asked if they had ever experienced calabar swelling (swelling under the skin that moves or disappears after a few days). The full questionnaire can be viewed in Supplemental Table 1. Smartphones, running the Secure Data Kit platform, were used to collect Global Positioning System (GPS) coordinates at every household location.

Everyone who was enrolled in the study was assigned a unique barcode ID and received a paper bracelet to wear with this unique ID. Participants were asked to go to a central location in the community between the hours of 10 am and 2 pm for diagnostic testing. The work of enrollment started early in the morning, to capitalize on the time of day when most individuals are at home and to ensure that there would be enough time for individuals to go to the central site for testing.

Informed consent was obtained from all adult participants and from parents or legal guardians of minors. Ethical approval for the study was obtained from the institutional review committee affiliated with the Ministry of Health of Gabon [approval no. #0254].

### Specimen collection and diagnostics.

Upon arrival at the central testing site, individuals progressed through a testing queue. After a finger prick blood draw, 20 µL of blood were collected directly into a calibrated capillary, which was then fed into the LoaScope. While the LoaScope reading was taking place, 10 µL of whole blood was collected to test for antifilarial antibodies against onchocerciasis (Ov16) and LF (Wb123) using the SD biplex rapid diagnostic test.^13^ Five microliters of blood was used to test for antibodies to the *L. loa* parasite, using the Loa antibody rapid diagnostic test. An additional 75 µL of blood was used to detect circulating filarial antigen via the Alere^™^ filariasis test strip (FTS). Finally, 60 µL of blood was collected onto TropBio filter papers, to preserve dried blood spots for future diagnostic testing. All diagnostics were performed using a single finger prick. For each of the tests mentioned, a laboratory technician reviewed the unique ID from the individual’s bracelet and a matching barcode label was then placed on the diagnostic test to enable linking of the results in the database. Each test was read at its prescribed time and the results were entered into the smartphones and communicated back to the participants in real time. Patients that tested positive by any diagnostic were not treated at the time of the study, but were instead advised to go to the peripheral health center for follow-up.

Positive controls were used to assess the performance of the FTS and SD biplex; no controls were available for the *L. loa* diagnostics.

### Loa antibody cutoff.

Given that this was only the second field validation of the new Loa antibody rapid test, it was necessary to establish a method for discriminating positive and negative results. A handheld reader was used to get a quantitative reading of the test results, based on the strength of the test line. A mixture model, using an expectation-maximization algorithm, was applied to these quantitative results and a cutoff of 157 reader units was deemed optimal and resulted in specificity of > 95% for other filarial infections (M. Biamonte, unpublished data).

### Geostatistical predictive maps.

Model-based geostatistics (MBG) was used to produce the predicted prevalence surface of the diseases. MBG methods have been widely used for prevalence mapping, especially in low resource settings where disease registries do not exist. The MBG approach has been previously described in detail.^14^ Briefly, the intuition behind the approach is as follows:

A prevalence survey conducted in a village at a geographical location *x* generates data in the form of a pair of values: *n*, the number of individuals surveyed; and *y*, the number of people that tested positive for the disease. The sampling distribution of *y* is binomial with number of trials *n* and probability of positive outcome *P*(*x*), the village-wide prevalence at *x*. The variation in *P*(*x*) over the region of interest can be explained by a combination of three phenomena: socioeconomic and environmental covariate effects *d*(*x*); unexplained residual spatial variation, *S*(*x*), which we model as a latent stochastic process; and unexplained residual nonspatial variation, *Z*, which we model as a set of independent and identically Gaussian distributed random effects. The standard way to account for these three components is with a logistic geospatial model,

log(P(x)/(1−P(x)) = d(x)β + S(x) + Z

The covariates used in the modeling were elevation and normalized difference vegetation index (NDVI). An advantage of the geospatial modeling is that it allows for prediction at unobserved locations while pooling information from neighboring locations, and so increases precision.^15^ Model parameters were estimated by maximum likelihood, using the R package PrevMap.^16^

## RESULTS

A total of 153 villages were visited and 7,751 individuals were sampled across the seven departments. In Boumi-Louetsi, one of the three departments selected for the joint mapping of LF, onchocerciasis, and loiasis, only 26 of the 30 selected villages were visited due to challenges with accessibility. In the other joint mapping departments, Mongo and Mpassa, all 30 selected villages were visited. The age of respondents ranged from 10 to 118 years old, with a mean of 41 years. Fifty-four percent of respondents were women, although the proportion of females in the sample varied by department, ranging from 48.5% in Mongo to 60% in Boumi-Louetsi (Table 1).

**Table 1:** Sample and demographic characteristics by department in Gabon

Department	Number of villages	Number of enrollees	Age (mean [min, max])	Proportion of females (%)
BOUMI-LOUETSI	26	1,341	43 (10, 100)	60.0
DOLA	22	1,287	40 (10, 97)	55.2
DOUIGNY	12	922	45 (10, 118)	56.5
DOUTSILA	12	685	43 (10, 109)	52.3
LEBOMBI-LEYOU	10	520	40 (10, 86)	57.3
MONGO	30	850	38 (10, 100)	48.5
MOUGOUTSI	11	493	47 (10, 98)	58.2
MPASSA	30	1,653	34 (10, 117)	49.8
Total	153	7,751	41 (10, 118)	54.4

### Onchocerciasis.

**Figure 2.**
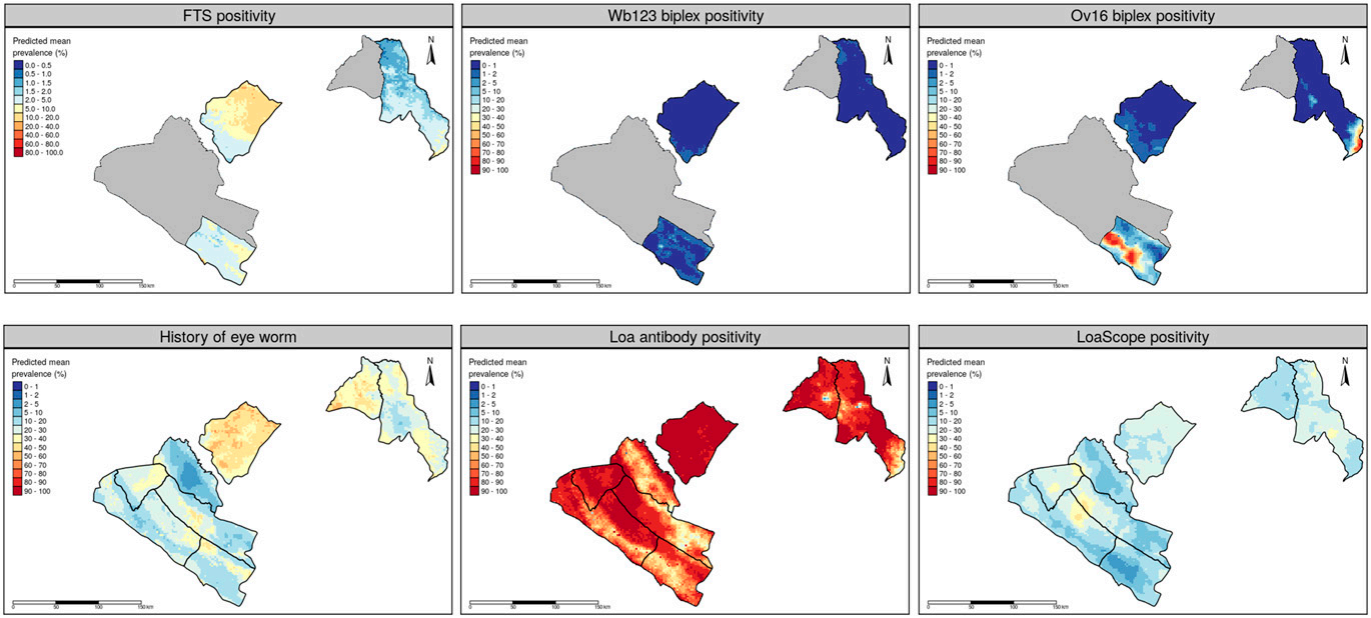
Predicted mean prevalence of indicators for lymphatic filariasis, onchocerciasis, and loiasis.

The mean department-wide prevalence of Ov16 antibodies ranged from 0.5% in Mpassa, to 1.2% in Boumi-Louetsi, to 39% in Mongo (Table 2). The greatest variation in village-level prevalence was seen in Mongo, which had villages with mean Ov16 prevalence ranging from 0% to 80%. The predicted prevalence map for onchocerciasis in the three departments, based on prevalence by Ov16 biplex rapid test, is shown in Figure 2. The maps indicate a wide variation in Ov16 prevalence within Mongo, with a high prevalence focus in the central portion of the department. In Mpassa, the maps indicate very little prevalence of Ov16, with the exception of the southern border with Republic of Congo, where prevalence is markedly greater. At the village level, there was a significant inverse relationship (Pearson’s *r* = −0.4084, *P* < 0.0001) between Ov16 prevalence and *L. Loa* mf prevalence.

**Table 2:** Department-wide mean prevalence of indicators for onchocerciasis, lymphatic filariasis, and loiasis, along with the minimum and maximum village-level prevalence

	Ov16 biplex positivity	Wb123 biplex positivity	Filariasis Test Strip positivity	Loa antibody rapid test positivity*		LoaScope prevalence	LoaScope prevalence of high intensity (> 20,000 mf/mL)	History of eye worm
Department	Department mean prevalence [village min, village max]	Department mean prevalence [village min, village max] %	Department mean prevalence [village min, village max]	Department mean prevalence [village min, village max]%	LoaScope intensity†‡ Mean [range]	Department mean prevalence [village min, village max]	Department mean prevalence [village min, village max]	Department mean prevalence [village min, village max]
BOUMI-LOUETSI	1.23 [0, 13.2]	0.46 [0, 4.55]	5.58 [0, 23.1]	88.0 [62.9, 100.0]	6,075 [150, 66,255]	24.7 [9.8, 51.1]	2.1 [0, 10.6]	39.1 [17.6, 100]
DOLA	NA	NA	NA	44.3 [24.7, 97.3]	5,229 [150, 41,240]	8.50 [0, 50.0]	0.8 [0, 7.9]	2.80 [0, 25.6]
DOUIGNY	NA	NA	NA	83.8 [50, 100]	5,789 [150, 60,000]	27.1 [12.9, 48.3]	2.3 [0, 6.3]	32.8 [18.5, 58.1]
DOUTSILA	NA	NA	NA	62.5 [33.3, 93.0]	5,825 [150, 50,694]	15.9 [8.2, 33.3]	1.3 [0, 16.7]	23.1 [10, 32.1]
LEBOMBI-LEYOU	NA	NA	NA	69.9 [37.0, 92.0]	2908 [150, 18042]	10.8 [2.9, 25.0]	0 [0, 0]	40.2 [19.4, 56.0]
MONGO	38.9 [0, 80.0]	2.29 [0, 56.2]	2.77 [0, 25.0]	55.2 [29.1, 100]	4,388 [150, 30,645]	5.44 [0, 50.0]	0.3 [0, 9.1]	16.9 [0, 51.9]
MOUGOUTSI	NA	NA	NA	78.1 [40.5, 100]	4,824 [150, 40,362]	29.5 [1.3, 72.7]	1.2 [0, 9.1]	15.4 [8.86, 30.0]
MPASSA	0.50 [0, 8.57]	0.12 [0, 2.17]	1.59 [0, 17.6]	55.5 [28.6, 96.4]	2,714 [150, 52,186]	19.6 [0, 53.8]	0.6 [0, 10.3]	18.7 [0.0, 53.8]
Total	13.5 [0, 80]	0.96 [0, 56.3]	3.31 [0, 25.0]	67.2 [24.7, 100]	4,719 [150, 66,225]	17.7 [0, 72.7]	1.1 [0, 16.7]	23.6 [0, 100]

* The cutoff for positivity was set at > 157 reader units.

† Analysis restricted to individuals testing positive by LoaScope.

‡ Lower threshold for valid LoaScope results is 150 mf/mL, based on previous field research.

### Lymphatic filariasis.

LF prevalence was measured by the presence of antifilarial antibodies (assessed by Wb123 biplex) and circulating filarial antigen (assessed by FTS). The mean department-wide prevalence of Wb123 antibodies ranged from 0.1% in Mpassa, to 0.5% in Boumi-Louetsi, to 2.3% in Mongo (Table 2). The greatest variation in village-level prevalence was seen in Mongo, which had villages with mean Wb123 prevalence ranging from 0% to 56.2%. FTS prevalence exceeded Wb123 prevalence at the department level, and ranged from 1.6% in Mpassa to 2.8% in Mongo and 5.6% in Boumi-Louetsi. The greatest village-level FTS prevalence was 25%, which was observed in Mongo. The predicted prevalence maps for LF indicate a very low prevalence with little heterogeneity by Wb123 biplex rapid test; however, the predictive maps for circulating filarial antigen suggest a more elevated prevalence with greater heterogeneity across the three departments (Figure 2). FTS and Wb213 positivity were not significantly correlated at the individual level (*P* = 0.176 by χ^2^ test) or at the village level (Pearson’s *r* = 0.073, *P* = 0.502), however, the low prevalence of both indicators likely prevents a statistically valid correlation assessment.

### Loiasis.

All the eight departments observed a high proportion of individuals testing positive for *L. loa* antibodies, with the department-wide mean ranging from 44.3% in Dola to 88% in Boumi-Louetsi (Table 2). At the village level, *L. loa* antibody prevalence ranged from 24.7% in Dola to 100% in Boumi-Louetsi, Mongo, and Mougoutsi. The mean department-wide prevalence of individuals with the *L. loa* parasite, as determined by the LoaScope, ranged from 5.4% in Mongo to 29.5% in Mougoutsi. Mougoutsi also had the maximum village-level prevalence detected by LoaScope, with 72.7% of adults testing positive in one village. Mean intensity of *L. loa* microfilaria ranged from 2,714 mf/mL in Mpassa to 6,075 in Boumi-Louetsi.

Prevalence of *L. loa* microfilariae was strongly associated with age across all departments (*P* < 0.001). Prevalence was lowest among those aged 10–14 and 15–19 (7.3% and 8.1%, respectively), and highest among those aged 40–49 (19.7%) and 50+ (25.5%). Results were similar among those aged 20–20 and 30–39 (14.0% and 13.9%). A similar trend was observed in the *L. loa* antibody results, with prevalence steadily increasing as age increased, from 39.1% among those aged 10–14 to 79.9% among those aged 50+.

Because individuals with > 20,000 *L. loa* mf/mL are at risk of SAEs when treated with IVM, we assessed the proportion of individuals in the sample with *L. loa* counts that exceeded this threshold. The prevalence of high-intensity loiasis infections was lowest in Lebombi-Leyou, where no one had an intensity > 20,000 mf/mL, whereas the greatest prevalence of high-intensity infections was observed in Douigny (2.3%). All the eight departments had at least one village where no one with a high-intensity infection was detected, whereas the maximum village level prevalence of high-intensity infections was seen in Doutsila, where 16.7% of individuals in a single village had high-intensity infections. History of eye worm was greatest in Lebombi-Leyou (mean prevalence of 40.2%) and lowest in Dola (mean prevalence of 2.8%).

The predictive prevalence maps for loiasis (mf, *L. loa* antibody and history of eye worm) all tell a slightly different story concerning the spatial variation of loiasis (Figure 2). The *L. loa* mf prevalence map indicates greatest heterogeneity in Mougoutsi and Doutsila, which share a high prevalence focus where the predicted mf prevalence is 50–60%. The *L. loa* antibody prevalence map indicates that *L. loa* antibodies, as assessed by the new rapid test, are readily detectable throughout the eight-department region. The prevalence of individuals with a history of eye worm appears to have greater spatial heterogeneity than *L. loa* mf, with the greatest predicted prevalence in Boumi-Louetsi and Lebombi-Leyou.

### Village-level results.

Results at the village level further indicate substantial variation in many of the indicators both within and across each department (Supplemental Table 2) that is illustrated in the maps shown in Figure 2. Only one of the villages in Mongo had an Ov16 prevalence of 0% (Moulengui Binza B), whereas the 29 other villages had a prevalence of at least 14%. Ov16 positivity was sparse in both Mpassa and Boumi-Louetsi, with prevalence detected in 7 of the 26 villages in Boumi-Louetsi and 3 of the 30 villages in Mpassa. Similarly, circulating filarial antigen was detected in all but 2 of the villages in Boumi-Louetsi (Haut Mbigou D and Kongui), but in just 9 and 12 of the 30 villages in Mpassa and Mongo, respectively. *Lola loa* microfilariae were detected in all but 14 of the 153 villages sampled, 9 of which were in Mongo. Among these 14 villages with 0% *L. loa* mf prevalence, 12 had *L. loa* antibody prevalence of 50% or greater, with one village in Mpassa (Sodato) having antibody prevalence as high as 92.3%.

### Joint distribution of LF and loiasis.

**Figure 3.**
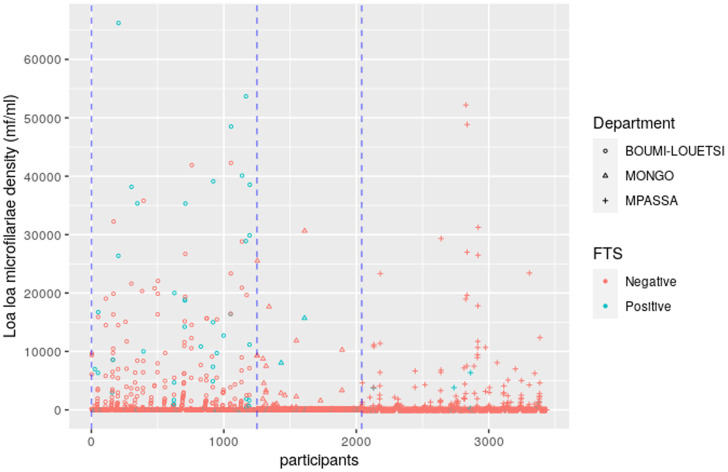
Joint plot of circulating filarial antigen status, assessed by filariasis test strip, and *Loa loa* microfilaria, among individuals tested for *L. loa* and lymphatic filariasis infection by department.

The joint distribution of individuals’ *L. loa* mf intensity (categorized as 0, 1–10,000, 10,001–20,000, 20,001–30,000, and > 30,000 mf/mL) by FTS result is presented for each of the three integrated mapping departments in Table 3. The data show a significant association between increasing *L. loa* mf intensity and FTS positivity for individuals living in Boumi-Louetsi (*P* < 0.001). This same association is not seen in Mongo or Mpassa, which may be the result of relatively low FTS positivity in both departments. A visual depiction of these same individual level results are shown by department in Figure 3.

**Table 3:** Joint distribution of circulating filarial antigen status, assessed by filariasis test strip, and intensity category of *Loa loa* microfilaria, among individuals tested for *L. loa* and lymphatic filariasis infection by department

	Boumi-Louetsi	Mongo	Mpassa	Total
LoaScope mf/mL	FTS Result*	FTS Result*	FTS Result*	FTS Result*
	[# pos./total (%)]	[# pos./total (%)]	[# pos./total (%)]	[# pos./total (%)]
0	39/306 (12.7%)	2/43 (4.7%)	6/271 (2.2%)	47/620 (7.6%)
1–10,000	16/248 (6.5%)	1/37 (2.7%)	6/253 (2.4%)	23/538 (4.3%)
10,001–20,000	10/33 (30.3%)	1/4 (25%)	0/10 (0%)	11/47 (23.4%)
20,001–30,000	4/12 (33.3%)	0/1 (0%)	0/5 (0%)	4/18 (22.2%)
> 30,000	9/13 (69.2%)	0/1 (0%)	0/3 (0%)	9/17 (52.9%)
Statistical significance^†^	< 0.001	0.386	0.979	< 0.001

FTS = filariasis test strip.

* Presented as the number of individuals testing FTS positive, divided by the total tested within the given category.

† Based on a chi-square test of association.

## DISCUSSION

This study presents the results from an integrated mapping assessment for onchocerciasis, LF, and loiasis. These findings demonstrate that a single population-based field survey can result in a wealth of information of programmatic relevance for all three diseases. The mapping data from Boumi-Louetsi, Mongo, and Mpassa indicate that all three diseases are endemic, albeit to varying degrees. The heterogeneity observed within each department for a given disease highlights the value of a mapping approach that utilizes cluster-based sampling, as opposed to relying on a limited number of purposeful sites. Results from this study confirmed that the decision by the Gabonese NTD program to not implement mass administration of IVM for onchocerciasis at the department level in the study area was appropriate because of the high prevalence of loiasis. This has led the NTD program to explore the possibility of alternative solutions—such as the “Test and (not) Treat” strategy using the LoaScope—that would allow for treatment determinations to be made at the individual level in departments (or subdepartments) in areas where the *L. loa* diagnostic results indicated that the risk of SAEs was high. In communities or subdepartments where the results indicated that the risk of SAEs was sufficiently low, the program may consider focalized mass treatment. Interestingly, the prevalence of onchocerciasis appears to be inversely related to the prevalence of *L. loa* mf at the village level. This is similar to the observations made by Kelly-Hope et al., who found contrasting spatial patterns in the prevalence of RAPLOA and onchocerca nodules in the Democratic Republic of Congo.^17^ While there clearly is overlap between the two vector species for onchocerciasis and loiasis—otherwise SAEs would not be a plausible concern—some of the difference in prevalence observed may be explained by differences in vector breeding habitats. *Simulium* species prefer fast flowing rivers, whereas *Chrysops* prefer swampy areas or muddy streams.

In addition to providing useful epidemiologic information across the three diseases, this study also generated information on the feasibility of such an integrated mapping approach. The methods facilitated successful collaboration between the administrative and health authorities of the regions and health departments, as well as within teams working in the field, to perform the study activities. The integrated approach also allowed for the development of expertise at the national level on utilizing new diagnostic technologies with the support of international consultants. Study teams were successfully able to implement the new technologies in the field following brief trainings. The activities also led to an updated database on filariasis prevalence in the study areas, as the study teams were able to achieve high population coverage in nearly all of the sampled villages due to the low population density in the study area.

This study provided important insights on the performance and programmatic utility of several diagnostic tests and demonstrated that it was possible to test for loiasis, onchocerciasis, and LF with one finger prick. In particular, this study represented one of the first field tests of the Loa antibody rapid test. The study teams used both visual analysis and a handheld reader to determine test positivity. Although the quantitative reader units were ultimately used to set a cutoff for positivity in this study, subsequent analysis of the data suggests that a qualitative reading, based on the presence or absence of the test line to the naked eye, may be sufficient for future determinations. In the time since this study was undertaken, field- and laboratory-based comparisons using the SD Wb123/Ov16 biplex have found the tool to be insufficiently sensitive for programmatic use when performed from whole blood.^11^ Consequently, though the presence of an Ov16 or Wb123 signal by the biplex is likely sufficient for detecting areas where MDA is warranted, the absence of a signal cannot be used to rule out areas where MDA is not necessary. For onchocerciasis, the current recommendation is to test for the presence of Ov16 antibodies using the biplex (or Ov16 monoplex) on eluated DBS,^11^ which is anticipated to occur in early 2022.

The cross-reactivity of FTS in individuals with high-intensity loiasis infections poses a challenge for programs seeking to map and monitor the prevalence of LF. Although we cannot provide a precise estimate on the true LF prevalence, we can estimate that the likely range in prevalence is between 1.80% (prevalence of FTS positivity among individuals free of *L. loa* mf) and 3.31% (all FTS positives). In the future, an LF antibody tool could replace the FTS (or serve as a confirmatory test) in *L. loa* endemic areas, as LF antibody tools have not been found to cross-react with high-intensity loiasis. However, in this study, there are concerns with the sensitivity of the Wb123 biplex result and thus this marker is unlikely to help rule out false FTS positives.

These methods and tools were subject to various limitations in the field. First, the LoaScope devices were prone to overheating (which was exacerbated by the hot, humid climate), particularly when used for prolonged periods in direct sunlight. This would cause the devices to slow down and become idle for 30 minutes after approximately 25 tests. This limitation did not, however, ultimately have a significant impact on the quality and quantity of the data collected. Slow Internet and data connection in the field also impacted the speed at which electronic data could be captured and uploaded. Additionally, accurate GPS coordinates were not always available at the household or village level due to malfunctions of the smartphones or because they were forbidden due to the site being a military camp. The unreliability of demographic data in the communities investigated made planning and resource allocation challenging. The number of devices needed per team and human resources required were underestimated, both of which led to prolonged study timelines. Sensitization activities in the selected communities proved to be critical, and in some cases did not adequately take place in the departments selected for the expanded loiasis testing. This led to low participation from some selected households due to sociocultural concerns. The lack of LF and onchocerciasis data from five of the eight departments prevented a complete picture of disease prevalence across all study sites. Finally, the deviation from the standard RAPLOA questionnaire (specifically the omission of the third question) precluded an exact one-to-one comparison to previously collected RAPLOA data.

It is important not to lose sight of why integrated mapping for onchocerciasis and loiasis is crucial for countries like Gabon. IVM treatment of onchocerciasis has been withheld from large populations where *L. loa* is prevalent and the risk of blindness is deemed to be low. This important safety precaution was necessary when it was impractical to predict where individuals are at risk of an SAE; however, with the LoaScope and *L. loa* antibody rapid test, as well as a statistical model to predict village-level risk, the tools now exist to predict where the risk of SAEs is sufficiently low. The remaining challenge is how to use these tools in combination to enable programs to make safe and efficient decisions regarding where to deliver MDA.

## Supplemental Material


Supplemental materials
