# Supplementary material for: An Integrated District Mapping Strategy for Loiasis to Enable Safe Mass Treatment for Onchocerciasis in Gabon
Source: Am J Trop Med Hyg. 2021 Nov 15;106(2):732–9. doi: 10.4269/ajtmh.21-0799 (PMC8832888; doi:10.4269/ajtmh.21-0799)
Supplement: Supplementary file 1 [file tpmd210799.SD1.pdf]

Supplemental File 1. Participant questionnaire

Question	Variable type	Response options
1. Enter the recorder ID number	integer	
2. Enter the survey type	select one	Phase 1; Phase 2
3. Enter community/village code	text	
4. Enter community/village code again	text	
5. Enter Household Identifier Code	Integer	
6. Is the subject eligible for screening today?	select one	Yes; No
7. If no, why was subject ineligible?	select one	Absent; Due to disability (severe illness, intellectual); Refused
8. If Absent: Will person likely attend the screening later today?	select one	Yes; No
9. Was proper consent/assent obtained?	select one	Yes; No
10. Select consent method	select one	Self; Parent/Guardian; Community leader
11. Enter last name	text	
12. Enter first name	text	
13. Select sex	select one	Female; Male
14. Enter age	integer	
15. Select method: barcode scan or manual entry	select one	Barcode; Manual
16. Scan barcode	barcode	
17. Enter barcode number	text	
18. Enter barcode number again	text	

19. How long have you lived in this community/village?	select one	10+ years; 5-9 years; 1-4 years; <1 year
20. Have you received treatment with Ivermectin and/or Albendazole?	select one	Yes; No; Unknown
21. Have you ever experienced or noticed worms move along the white of your eye?	select one	Yes; No
22. Have you ever had the condition in this picture? (show picture of eyeworm)	select one	Yes; No
23. If yes, how many times have you experienced this over the past year?	integer	
24. Have you ever experienced swellings under the skin that change position or disappear (Calabar swelling)?	select one	Yes; No
25. If yes, on what part(s) of your body did the swelling occur?	text	
26. If yes, for how long did the swelling last (days)?	integer	
27. Do you know any persons in this locality who had this type of swelling?	select one	Yes; No
