# Supplementary material for: An Integrated District Mapping Strategy for Loiasis to Enable Safe Mass Treatment for Onchocerciasis in Gabon
Source: Am J Trop Med Hyg. 2021 Nov 15;106(2):732–9. doi: 10.4269/ajtmh.21-0799 (PMC8832888; doi:10.4269/ajtmh.21-0799)
Supplement: Supplementary file 2 [file tpmd210799.SD2.pdf]

Supplemental Table 1. Village-level prevalence of indicators for onchocerciasis, lymphatic filariasis and loiasis

S/N	Department	Village name	Ov16 biplex positivity	Wb123 biplex positivity	Filariasis Test Strip positivity	Loa antibody positivity[1]	LoaScope intensity[2, 3]	LoaScope prevalence	LoaScope prevalence of high intensity (>20,000 mf/ml)	History of eye worm
			Village prevalence (%)	*Village prevalence (%)*	*Village prevalence (%)*	*Village prevalence (%)*	*Village mean intensity*	*Village prevalence (%)*	*Village prevalence (%)*	*Village prevalence (%)*
1	BOUMI-LOUETSI	HAUT MBIGOU A	0.00	0.00	8.33	75.00	6311	16.00	0.00	28.00
2	BOUMI-LOUETSI	HAUT MBIGOU B	0.00	0.00	21.43	75.00	3834	14.29	0.00	21.43
3	BOUMI-LOUETSI	HAUT MBIGOU C	0.00	1.52	7.58	81.54	3795	21.21	0.00	32.61
4	BOUMI-LOUETSI	HAUT MBIGOU D	0.00	0.00	0.00	80.49	6682	16.67	0.00	23.81
5	BOUMI-LOUETSI	NZENZELE A	2.22	0.00	4.44	97.78	5930	51.11	2.22	66.67
6	BOUMI-LOUETSI	NZENZELE B	0.00	0.00	2.94	95.77	8184	25.00	2.78	50.00
7	BOUMI-LOUETSI	NZENZELE C	0.00	0.00	3.03	96.92	4764	30.30	3.03	80.30
8	BOUMI-LOUETSI	MOUNGUEMBE A	1.49	0.00	6.67	85.07	6962	20.90	2.99	39.71
9	BOUMI-LOUETSI	MOUNGUEMBE B	0.00	0.00	3.13	96.83	6853	15.87	1.59	60.29
10	BOUMI-LOUETSI	MAKONGONIO A	0.00	0.00	3.57	92.59	1349	17.86	0.00	64.29
11	BOUMI-LOUETSI	MAKONGONIO B	0.00	0.00	4.76	95.00	9691	14.29	4.76	42.86
12	BOUMI-LOUETSI	BAS BOUMI A	1.89	1.89	3.92	79.25	9963	15.09	1.89	43.40
13	BOUMI-LOUETSI	BAS-BOUMI B	1.54	0.00	1.54	78.46	2667	15.38	0.00	36.36

14	BOUMI-LOUETSI	COMMERCIAL	0.00	0.00	12.70	62.90	2721	23.44	1.56	39.06
15	BOUMI-LOUETSI	LEKINDOU	0.00	1.32	5.41	98.68	6780	48.05	3.90	58.44
16	BOUMI-LOUETSI	MOUKOUNDZA	0.00	0.00	1.04	70.21	8525	13.54	1.04	39.58
17	BOUMI-LOUETSI	MANDJI	13.24	1.47	1.47	98.48	2521	39.71	0.00	69.57
18	BOUMI-LOUETSI	IMENO MBILA	0.00	0.00	18.52	92.31	10369	25.93	3.70	48.15
19	BOUMI-LOUETSI	LEDOUME	0.00	0.00	6.12	89.09	3721	19.23	0.00	47.27
20	BOUMI-LOUETSI	MAYANI	0.00	0.00	1.89	98.41	3938	20.97	1.61	48.44
21	BOUMI-LOUETSI	ISSALA	2.33	0.00	5.00	95.24	8992	41.86	6.98	67.44
22	BOUMI-LOUETSI	DIBWANGUI	0.00	0.00	7.14	100.00	1848	9.76	0.00	50.00
23	BOUMI-LOUETSI	LEPOYE	4.26	0.00	6.38	100.00	10503	40.43	10.64	70.00
24	BOUMI-LOUETSI	KAMBAMONGO	0.00	4.55	23.08	88.89	4398	31.82	0.00	36.36
25	BOUMI-LOUETSI	KONGUI	0.00	0.00	0.00	100.00	768	25.00	0.00	100.00
26	BOUMI-LOUETSI	BOUTSANA	0.00	1.54	13.85	81.97	8383	17.19	3.13	35.29
27	DOLA	NYANGA YOUNGOU	NA	NA	NA	97.30	7824	50.00	7.89	25.64
28	DOLA	AVIATION	NA	NA	NA	42.42	319	4.48	0.00	5.97
29	DOLA	MOUNIODJI	NA	NA	NA	63.33	7043	13.33	0.00	10.00
30	DOLA	FERA	NA	NA	NA	43.40	9821	7.27	1.82	0.00
31	DOLA	MBADI	NA	NA	NA	51.85	NA	0.00	0.00	0.00
32	DOLA	DILOLO	NA	NA	NA	72.73	2237	4.55	0.00	0.00
33	DOLA	TSAGUI	NA	NA	NA	64.71	NA	0.00	0.00	5.88
34	DOLA	MOUSSAMBOU	NA	NA	NA	85.71	2567	24.00	2.00	1.96

35	DOLA	SAINT JOSELH	NA	NA	NA	37.04	NA	0.00	0.00	3.70
36	DOLA	MAPEMBI	NA	NA	NA	41.38	5625	6.67	1.11	0.00
37	DOLA	DOUSSALA	NA	NA	NA	47.44	3098	13.92	0.00	2.53
38	DOLA	SAINT PAUL	NA	NA	NA	39.66	2971	10.00	0.00	6.45
39	DOLA	MAPENDI	NA	NA	NA	26.92	1908	2.00	0.00	5.66
40	DOLA	LAC BLEU	NA	NA	NA	40.58	9504	5.80	1.45	4.29
41	DOLA	MOUNIONDZI	NA	NA	NA	52.56	1283	8.86	0.00	0.00
42	DOLA	MALABA2	NA	NA	NA	39.47	229	5.26	0.00	0.00
43	DOLA	MONTAGNE SAINTE	NA	NA	NA	34.41	5725	1.03	0.00	0.00
44	DOLA	CAMP TP	NA	NA	NA	40.82	1725	8.00	0.00	4.00
45	DOLA	POTO POTO	NA	NA	NA	35.45	6117	5.31	0.00	0.85
46	DOLA	MALABA1	NA	NA	NA	30.61	658	3.92	0.00	1.96
47	DOLA	SAINT PIERRE	NA	NA	NA	42.68	9435	10.98	2.44	0.00
48	DOLA	MINDANDA	NA	NA	NA	24.68	7337	7.59	1.27	0.00
49	DOUIGNY	MOCABE	NA	NA	NA	92.86	7529	37.80	4.88	36.59
50	DOUIGNY	MURINDI	NA	NA	NA	95.00	6773	34.38	4.69	18.46
51	DOUIGNY	POUTOUNENI	NA	NA	NA	86.00	7876	32.26	3.23	27.42
52	DOUIGNY	MOUTOUMBA	NA	NA	NA	66.00	8392	12.90	2.15	20.43
53	DOUIGNY	MBAMBA	NA	NA	NA	82.35	4274	23.53	0.00	28.99
54	DOUIGNY	MOUDIBA	NA	NA	NA	68.75	3055	17.00	0.00	28.00
55	DOUIGNY	DJABA	NA	NA	NA	100.00	2507	21.18	0.00	30.59

56	DOUIGNY	MIAMBA	NA	NA	NA	50.00	3943	14.00	0.00	27.00
57	DOUIGNY	MISSAFOU	NA	NA	NA	84.09	5247	25.51	2.04	34.00
58	DOUIGNY	BILENGUI	NA	NA	NA	100.00	5891	43.75	6.25	52.08
59	DOUIGNY	MOUKOKO MBAKA	NA	NA	NA	100.00	6834	48.39	4.84	59.68
60	DOUIGNY	MOUGOUNA	NA	NA	NA	85.19	5206	40.74	3.70	51.79
61	DOUTSILA	DOUANO 2	NA	NA	NA	93.02	7245	23.26	2.33	24.44
62	DOUTSILA	DOUVOULI	NA	NA	NA	62.00	4241	14.00	0.00	10.00
63	DOUTSILA	NYALI	NA	NA	NA	92.73	9072	26.79	3.57	32.14
64	DOUTSILA	PANZA 2	NA	NA	NA	62.00	10306	22.00	4.00	24.00
65	DOUTSILA	BANDA MAMBA	NA	NA	NA	72.50	1298	12.50	0.00	17.50
66	DOUTSILA	KOTA	NA	NA	NA	36.84	984	10.53	0.00	10.53
67	DOUTSILA	KOUMOU DOUSSALA	NA	NA	NA	33.33	13645	33.33	16.67	16.67
68	DOUTSILA	BAMBOMA	NA	NA	NA	55.00	4476	20.00	1.00	24.00
69	DOUTSILA	DONGO	NA	NA	NA	71.74	3819	12.22	0.00	24.44
70	DOUTSILA	DOUDZANZA	NA	NA	NA	45.36	2156	9.18	0.00	26.53
71	DOUTSILA	NGONGO	NA	NA	NA	60.76	4370	15.00	1.25	25.00
72	DOUTSILA	NZINGA	NA	NA	NA	51.02	9609	8.16	2.04	19.61
73	LEBOMBI-LEYOU	DJOUTOU	NA	NA	NA	75.00	1512	10.42	0.00	52.08
74	LEBOMBI-LEYOU	LEKAMBA	NA	NA	NA	48.00	1448	5.56	0.00	19.44
75	LEBOMBI-LEYOU	NDJIMA	NA	NA	NA	76.47	2302	16.67	0.00	56.00
76	LEBOMBI-LEYOU	LEMAGNA	NA	NA	NA	82.19	3024	8.64	0.00	27.16

77	LEBOMBI-LEYOU	IDEMBE	NA	NA	NA	36.96	5374	8.33	0.00	44.26
78	LEBOMBI-LEYOU	NGUIASSONO	NA	NA	NA	91.67	1710	25.00	0.00	36.11
79	LEBOMBI-LEYOU	IYEYE	NA	NA	NA	50.00	150	2.94	0.00	50.00
80	LEBOMBI-LEYOU	MASSANGO 1	NA	NA	NA	73.21	1453	8.20	0.00	38.71
81	LEBOMBI-LEYOU	IDJIBA	NA	NA	NA	81.13	5626	10.61	0.00	40.91
82	LEBOMBI-LEYOU	KONDA	NA	NA	NA	76.09	2999	15.49	0.00	47.89
83	MONGO	BAYADI	45.24	0.00	2.38	78.57	11059	9.52	2.38	19.05
84	MONGO	BIKAMBA	30.00	0.00	0.00	90.00	2287	50.00	0.00	8.33
85	MONGO	DOUKI	71.43	0.00	0.00	57.14	4563	7.14	0.00	28.57
86	MONGO	GNOUMBITSI	37.04	3.70	11.11	NA	3713	15.38	0.00	55.56
87	MONGO	IKOLI 1	25.93	0.00	0.00	59.26	17658	3.70	0.00	29.63
88	MONGO	MAGANDI A	16.67	5.56	5.56	76.47	491	16.67	0.00	5.26
89	MONGO	MAGANDI B	25.00	8.33	0.00	100.00	648	16.67	0.00	16.67
90	MONGO	MIASSA	33.33	0.00	0.00	NA	1031	5.56	0.00	22.22
91	MONGO	BIBORA A	33.33	26.67	0.00	57.14	329	6.67	0.00	40.00
92	MONGO	BIBORA B	56.25	56.25	6.25	93.33	5463	12.50	0.00	6.25
93	MONGO	DUBONAIRE	66.67	0.00	0.00	54.55	NA	0.00	0.00	33.33
94	MONGO	DILEMBA A	18.87	0.00	0.00	64.15	1376	3.85	0.00	22.64
95	MONGO	DILEMBA B	17.50	0.00	2.50	72.50	284	6.06	0.00	10.00
96	MONGO	DILEMBA C	18.18	0.00	7.27	49.02	3679	8.51	0.00	20.00
97	MONGO	DOUBARA 1	41.18	0.00	0.00	76.47	NA	0.00	0.00	29.41

98	MONGO	MOULENGUI BINZA A	18.18	0.00	9.09	81.82	23176	18.18	9.09	9.09
99	MONGO	MOULENGUI BINZA B	0.00	0.00	25.00	50.00	NA	0.00	0.00	0.00
100	MONGO	RINAZALA A	29.73	0.00	5.41	60.53	154	2.70	0.00	7.14
101	MONGO	RINAZALA B	19.23	0.00	0.00	45.83	150	3.85	0.00	5.56
102	MONGO	DOUMANGA VILLAGE	65.91	0.00	0.00	45.45	NA	0.00	0.00	20.45
103	MONGO	NOM INDéTERMINéE 1A	38.10	0.00	0.00	61.90	1579	4.76	0.00	19.05
104	MONGO	NOM INDéTERMINéE 1B	40.00	0.00	0.00	60.00	NA	0.00	0.00	0.00
105	MONGO	NOM INDÉTERMINÉE 1C	80.00	0.00	0.00	57.14	NA	0.00	0.00	40.00
106	MONGO	NOM INDéTERMINéE 2	24.14	0.00	0.00	50.00	NA	0.00	0.00	8.62
107	MONGO	DOUMANGA CENTRE A	50.62	2.47	1.23	29.11	197	1.25	0.00	7.41
108	MONGO	DOUMANGA CENTRE B	48.89	0.00	1.11	41.11	6800	2.33	0.00	21.11
109	MONGO	VOUNGOU	50.00	0.00	0.00	50.00	NA	0.00	0.00	50.00
110	MONGO	MAGONGA	68.89	0.00	11.11	31.82	241	2.22	0.00	6.52
111	MONGO	INDÉTERMINÉ 3	14.29	0.00	0.00	57.14	NA	0.00	0.00	0.00
112	MONGO	INDÉTERMINÉ 4	55.56	11.11	11.11	88.89	295	22.22	0.00	11.11
113	MOUGOUTSI	MABOURIGHA	NA	NA	NA	100.00	6833	72.73	0.00	27.27
114	MOUGOUTSI	MANDILOU 02	NA	NA	NA	NA	3758	58.06	0.00	21.88
115	MOUGOUTSI	NDENGUILILA	NA	NA	NA	93.98	4709	50.60	3.61	18.60
116	MOUGOUTSI	PENIOUNDOU	NA	NA	NA	NA	3989	28.89	0.00	8.89

117	MOUGOUTSI	TONO VILLAGE	NA	NA	NA	88.10	4554	35.71	2.38	11.90
118	MOUGOUTSI	BIBORA	NA	NA	NA	NA	3301	8.51	0.00	17.02
119	MOUGOUTSI	NDABILILA	NA	NA	NA	NA	4206	14.49	0.00	9.46
120	MOUGOUTSI	CHATEAU	NA	NA	NA	40.51	2172	1.28	0.00	8.86
121	MOUGOUTSI	MAKABANA	NA	NA	NA	93.33	5064	45.45	0.00	26.67
122	MOUGOUTSI	DOUSSEOUSSOU	NA	NA	NA	70.00	13600	10.00	0.00	30.00
123	MOUGOUTSI	MASSIEGA VILLAGE	NA	NA	NA	95.45	7239	45.45	9.09	18.18
124	MPASSA	EBALA	0.00	0.00	0.00	48.39	1279	9.84	0.00	20.90
125	MPASSA	EYOUGA 2	0.00	2.17	5.13	71.74	1185	6.67	0.00	50.00
126	MPASSA	MBOUMA OYALI	0.00	1.79	9.26	71.43	4380	18.92	0.00	42.86
127	MPASSA	NDOUNGOU	0.00	0.00	0.00	96.36	2860	40.43	2.13	26.32
128	MPASSA	BASE AéRIENNE 02	0.00	0.00	0.00	38.46	215	1.28	0.00	0.00
129	MPASSA	MVENGUE VILLAGE B	0.00	0.00	3.85	31.37	557	53.85	0.00	33.96
130	MPASSA	CAMP DJOUé DABANY	0.00	0.00	0.00	41.00	486	3.00	0.00	0.00
131	MPASSA	MVENGUE VILLAGE A	0.00	0.00	0.00	55.71	2284	7.14	0.00	2.70
132	MPASSA	SUCAF GABON A	0.00	0.00	0.00	35.19	744	21.28	0.00	25.93
133	MPASSA	SUCAF GABON B	0.00	0.00	0.00	42.47	575	5.41	0.00	3.75
134	MPASSA	SUCAF GABONC	0.00	0.00	0.00	34.15	5132	12.35	1.23	5.95
135	MPASSA	SUCAF GABON D	6.25	0.00	7.81	49.21	911	32.56	0.00	18.46
136	MPASSA	OUELE SUCAF	0.00	0.00	1.56	33.87	743	21.05	0.00	19.40
137	MPASSA	MOTOBO2	0.00	0.00	0.00	86.36	2969	46.15	0.00	31.82

138	MPASSA	EBORI	0.00	0.00	0.00	95.92	25277	24.14	10.34	20.00
139	MPASSA	SODATO	0.00	0.00	0.00	92.31	NA	0.00	0.00	53.85
140	MPASSA	MVOUNA 2 PONTS B	0.00	0.00	17.65	81.82	2662	15.00	0.00	34.09
141	MPASSA	POUBARA	0.00	0.00	0.00	62.50	2830	6.25	0.00	0.00
142	MPASSA	MOUPIA	0.00	0.00	0.00	92.50	9247	20.31	3.13	2.41
143	MPASSA	BENGUIA 2	0.00	0.00	0.00	56.06	1339	28.79	0.00	25.00
144	MPASSA	GENDARMERIE	0.00	0.00	0.00	57.14	206	15.00	0.00	42.86
145	MPASSA	SUCAF GABON E	0.00	0.00	0.00	60.00	NA	0.00	0.00	20.00
146	MPASSA	SUCAF GABON F	0.00	0.00	0.00	41.67	8072	2.78	0.00	2.78
147	MPASSA	CAMP SINO HYDRO	8.57	0.00	2.86	28.57	879	48.48	0.00	16.67
148	MPASSA	SUCAF A	1.75	0.00	1.75	46.43	1052	42.86	0.00	34.48
149	MPASSA	SUCAF GABON B	0.00	0.00	0.00	38.89	3574	3.70	0.00	9.09
150	MPASSA	SUCAF C	0.00	0.00	0.00	51.28	749	39.13	0.00	31.33
151	MPASSA	LEKOUSSAGA	0.00	0.00	0.00	92.00	7259	26.92	3.85	23.08
152	MPASSA	MVOUNA A	0.00	0.00	0.00	85.96	4614	14.04	1.75	2.94
153	MPASSA	BASE AÉRIENNE 02 B/ MAPOUBA	0.00	0.00	2.50	44.87	1404	35.06	0.00	40.00
Total			13.5	0.96	3.31	67.2	4719	17.7	1.1	23.6

NA – Data not collected

[1] The cutoff for positivity was set at >157 reader units.
[2] Analysis restricted to individuals testing positive by LoaScope
[3] Lower threshold for valid LoaScope results is 150 mf/mL, based on previous field research
